# Immunological and genetic kinetics from diagnosis to clinical progression in chronic lymphocytic leukemia

**DOI:** 10.1186/s40364-021-00290-z

**Published:** 2021-05-20

**Authors:** Isabel Jiménez, Bárbara Tazón-Vega, Pau Abrisqueta, Juan C. Nieto, Sabela Bobillo, Carles Palacio-García, Júlia Carabia, Rafael Valdés-Mas, Magdalena Munuera, Lluís Puigdefàbregas, Genís Parra, Anna Esteve-Codina, Clara Franco-Jarava, Gloria Iacoboni, María José Terol, José Antonio García-Marco, Marta Crespo, Francesc Bosch

**Affiliations:** 1 Experimental Hematology, Vall d’Hebron Institute of Oncology (VHIO), Vall d’Hebron Barcelona Hospital Campus, C/Natzaret 115-117, 08035 Barcelona, Spain; 2 Department de Medicina, Universitat Autònoma de Barcelona, 08193 Bellaterra, Spain; 3 Servei d’Hematologia, Vall d’Hebron Hospital Universitari, Experimental Hematology, Vall d’Hebron Institute of Oncology (VHIO), Vall d’Hebron Barcelona Hospital Campus, Passeig Vall d’Hebron 119-129, 08035 Barcelona, Spain; 4 DREAMgenics, 33011 Oviedo, Spain; 5 Centre for Genomic Regulation, Barcelona Institute of Science and Technology, 08003 Barcelona, Spain; 6 Universitat Pompeu Fabra, 08002 Barcelona, Spain; 7 Servei d’Immunologia, Vall d’Hebron Institut de Recerca (VHIR), Vall d’Hebron Hospital Universitari, Vall d’Hebron Barcelona Hospital Campus, Passeig Vall d’Hebron 119-129, 08035 Barcelona, Spain; 8 Department of Hematology, Clínic University Hospital, INCLIVA Biomedical Research Institute, 46010 Valencia, Spain; 9 Department of Hematology, Puerta de Hierro University Hospital, 28222, Majadahonda, Madrid, Spain

**Keywords:** CLL, Immune evasion, Clinical progression, T cell exhaustion

## Abstract

**Background:**

Mechanisms driving the progression of chronic lymphocytic leukemia (CLL) from its early stages are not fully understood. The acquisition of molecular changes at the time of progression has been observed in a small fraction of patients, suggesting that CLL progression is not mainly driven by dynamic clonal evolution. In order to shed light on mechanisms that lead to CLL progression, we investigated longitudinal changes in both the genetic and immunological scenarios.

**Methods:**

We performed genetic and immunological longitudinal analysis using paired primary samples from untreated CLL patients that underwent clinical progression (sampling at diagnosis and progression) and from patients with stable disease (sampling at diagnosis and at long-term asymptomatic follow-up).

**Results:**

Molecular analysis showed limited and non-recurrent molecular changes at progression, indicating that clonal evolution is not the main driver of clinical progression. Our analysis of the immune kinetics found an increasingly dysfunctional CD8^+^ T cell compartment in progressing patients that was not observed in those patients that remained asymptomatic. Specifically, terminally exhausted effector CD8^+^ T cells (T-bet^dim/−^Eomes^hi^PD1^hi^) accumulated, while the the co-expression of inhibitory receptors (PD1, CD244 and CD160) increased, along with an altered gene expression profile in T cells only in those patients that progressed. In addition, malignant cells from patients at clinical progression showed enhanced capacity to induce exhaustion-related markers in CD8^+^ T cells ex vivo mainly through a mechanism dependent on soluble factors including IL-10.

**Conclusions:**

Altogether, we demonstrate that the interaction with the immune microenvironment plays a key role in clinical progression in CLL, thereby providing a rationale for the use of early immunotherapeutic intervention.

**Supplementary Information:**

The online version contains supplementary material available at 10.1186/s40364-021-00290-z.

## Background

Identification of patients that are at high risk of progression to advanced clinical stages needing treatment in chronic lymphocytic leukemia (CLL) is expedited by the use of different prognostic parameters including the mutational status of the IGHV genes or its surrogates like ZAP-70 expression [1, 2], chromosomal aberrations and gene mutations (i.e., *TP53* or *NOTCH1*), [3, 4] or prognostic scores like the CLL-IPI [5]. Despite the great advances in the understanding of the biology of this disease over the last decade, the underlying mechanisms that drive clinical progression from early stages have not been fully deciphered yet.. Longitudinal genetic studies performed at the time of diagnosis and at progression before treatment show non-recurrent and limited changes in around half of the patients [6–15]. In agreement, changes in the gene expression profile of leukemic cells are also infrequent at progression [16]. These findings indicate that CLL progression from early stages is not mainly driven by genetic evolution, which highlights the potential role of the leukemic microenvironment in the evolution of the disease.

Besides the generalized immune dysfunction observed in patients diagnosed with CLL [17], circulating T cells from these patients exhibit an increased proportion of dysfunctional and exhausted antigen-experienced cells characterized by high expression of inhibitory receptors, impaired proliferation and cytotoxic activity as well as a defective immune synapse with leukemic cells, [18–22] all indicating defective anti-tumor T cell responses. Moreover, the immunosuppressing microenvironment prevalent in CLL is also boosted by the expansion of myeloid-derived suppressor cells (MDSCs) [23] and the production of IL-10 by CLL cells [24], among other factors.

These observations led us to hypothesize that a disease-induced T cell exhaustion accumulating from diagnosis could translate into an escape from immune surveillance driving CLL from early asymptomatic stages to clinical progression. To identify mechanisms of clinical progression we studied the specific changes that occur in patients that progress compared to those that remain asymptomatic along time. In detail, we performed genetic and immunological longitudinal analysis using paired primary samples from untreated CLL patients that experienced clinical progression (sampling at diagnosis and at progression) and from patients with stable disease (sampling at diagnosis and at long-term asymptomatic follow-up). Our results show that CLL cells exhibit limited and non-recurrent genetic changes at progression while CD8^+^ T cells display increased exhaustion features, potentially induced by IL-10 secreted by malignant B cells, and a differential transcriptome at progression. In contrast, patients without evidence of progression did not experience significant changes over time in their CD8^+^ T cell exhaustion status and the capacity of malignant cells to induce T cell exhaustion and to secrete IL-10 was lower compared to malignant cells from progressing patients and maintained over time.. These results could contribute to lay the foundations for the clinical testing of early therapeutic interventions on the immune system to prevent or delay progression of the disease.

## Methods

### Primary samples

Thirty eight patients diagnosed with CLL were enrolled in the study. Peripheral blood mononuclear cells (PBMCs) were isolated by Ficoll density gradient and cryopreserved. Plasma was obtained from EDTA blood and stored at − 80 °C. Samples were collected at two time points: diagnosis and progression before treatment or long-term asymptomatic follow-up. For most of the experiments, only a subgroup of the patients is represented due to availability of samples. For co-culture assays, PBMCs from age-matched healthy donors were used (HD; *n* = 17; 64 years old). A written informed consent was obtained from all individuals in accordance with the declaration of Helsinki and the study was approved by the local clinical research ethics committee.

### Whole-exome sequencing (WES) and data processing

Libraries for WES were sequenced on HiSeq2500 (Illumina, San Diego, CA, USA) with a read length of 100 bp paired-end. Raw data was processed using the Real Time Analysis software (RTA 1.18.66.3, Illumina) to generate FASTQ sequence files, which were processed using the bioinformatics software HD Genome One (DREAMgenics, Oviedo, Spain).

The detection of copy number variations (CNVs) was performed by a modified version of the exome2cnv algorithm [25]. For tumor samples, the algorithm employed a pool of all control samples as background. The cancer cell fraction (CCF) and the 95% confidence interval (CI) were computed using the R package Palimpsest [26]. See Supplementary Methods for detailed information.

### RNA sequencing (RNA-Seq) and data processing

Libraries for RNA-Seq were sequenced on HiSeq2500 (Illumina) with a read length of 100 bp paired-end. Reads were mapped against the human reference genome (GENCODE release 28) and genes were quantified as detailed in Supplementary Methods.

### Flow cytometry analysis

Cryopreserved PBMCs were thawed and stained with monoclonal antibodies (mAbs, Supplementary Table S1) as detailed in Supplementary Methods.

For the assessment of intracellular IL-10 produced by CLL cells, PBMCs were co-cultured for 48 h with UE6E7T-2 cells, CD40L (Peprotech, Rocky Hill, NJ, USA) and TLR9L (CpG ODN2006, Invivogen, San Diego, CA, USA) as we previously described [27] and then cells were stimulated with Leukocyte Activation Cocktail (BD Biosciences, Franklin Lakes, NJ, USA) for 5 h prior to staining.

Cells were acquired by a Navios™ cytometer (Beckman Coulter, Brea, CA, USA). Data were analyzed using the FlowJo v10 software (TreeStar, Ashland, OR, USA) and the Cytobank platform (Santa Clara, CA, USA).

### Determination of IL-10 in plasma

Concentrations of plasmatic IL-10 were measured using the Simple Plex™ Assay for the detection of human IL-10 (R&D Systems, Minneapolis, MN, USA) on Ella™ Automated ELISA Platform following the manufacturer’s instructions.

### B and T lymphocytes co-cultures

After negative selection, B-CLL cells or B cells from HD were co-cultured with T cells from CLL or HD at 1:2 and 1:10 T to B cell ratios as detailed in Supplementary Methods.

### Statistical analysis

Comparisons were performed using the Wilcoxon matched-pairs rank test or the Mann-Whitney U test in unpaired samples. Differences were considered statistically significant if *P* < 0.05. All the statistical analyses were carried out and graphed using the GraphPad Prism version 6.0 software.

## Results

### CLL cells show limited and non-recurrent genetic changes at clinical progression

In a series of 25 patients (median age: 63 years, range 40–82 years) that experienced clinical progression (median time to progression (TTP): 29 months, range 5–96 months), we collected serial paired samples at diagnosis and at the time of progression before treatment. Definition of progression and requirement for treatment were established following the international workshop on CLL (IWCLL) criteria [28]. As a control group, we collected serial samples at diagnosis and at follow-up from 13 patients that had stable disease (median age: 66 years, range 47–81 years; median time to second sampling: 39 months, range 30–77 months). For this group, the median follow-up was 77 months (range 41–101 months) and only one patient (CLL46) progressed (19 months after second sampling). Clinical characteristics are summarized in Table 1 and detailed in Supplementary Table S2.

**Table 1:** Clinical characteristics of progressing and non-progressing patients

	**Progressed**		**Non-progressed**	
Gender	M (68%)		M (69%)	
BINET/RAI stage at diagnosis	A0 (72%)		A0 (92%)	
IGHV Status	UM (56%)		UM (8%)	
	**Median**	**Range**	**Median**	**Range**
Age at diagnosis	63	40–82	66	47–81
TTP (months)	29	5–96	–	–
Follow-up without progression (months)	–	–	77	41–101
Time to second sampling (months)	29	5–96	39	30–77
Lymphocytes·10^9^/L at diagnosis	12.20	3.3-65.8	10.40	3.8-31.2
Lymphocytes·10^9^/L at second sampling	73.05	2.3-287.1	22.40	5.2-85.3

**Fig. 1:**
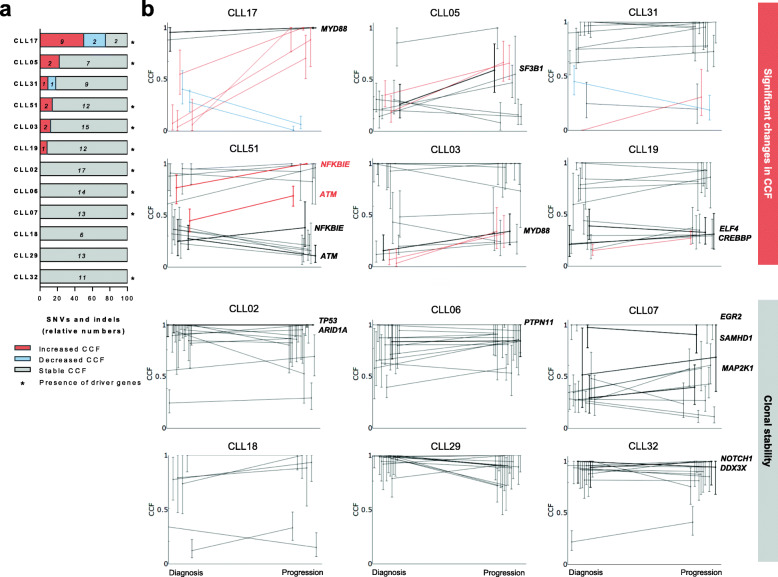
Longitudinal analysis of the CCF of SNVs and indels from paired B-CLL cells at diagnosis and progression before treatment. **a** Percentage of SNVs and indels with significantly increased (red),decreased (blue) or stable (grey) CCF from diagnosis to progression. Patients harboring mutations in CLL driver genes are marked with an asterisk. Absolute numbers of SNVs and indels detected per patient are detailed in italics inside bars. **b** Comparison of the CCF with 95% CI for each alteration detected per patient (*n* = 12) between diagnosis and progression. Significantly increased (red lines), decreased (blue lines) or stable CCF (grey lines) are shown. CLL driver genes are plotted with bold lines and labeled with gene name

For the purpose of analyzing potential genetic evolution related to clinical progression, we performed longitudinal WES in paired samples from 12 patients at diagnosis and progression. With a mean read depth of 110X, the limit of detection was set at a minimum coverage of 20 reads and a minimum of 0.1 variant allele frequency (VAF). In accordance with previous WES studies in CLL, mutation rate at both time points was low and consisted of a mean ± SEM (standard error of the mean) of 12.2 ± 3.3 (range 6–17) somatic single nucleotide variants (SNVs) and insertions and deletions (indels) per exome. (Supplementary Table S3). We then screened for clonal shifts from diagnosis to progression by calculating significant changes in the CCF of the alterations detected in each patient [29] (see supplementary methods for detailed information about CCF calculation). A significant change was determined if the 95% confidence intervals of the CCF in the diagnosis and progression sample did not overlap [29]. We found that 50% of patients showed significant changes at progression affecting the CCF of at least one alteration. However, the remaining 50% of patients exhibited clonal stability at clinical progression (Fig. 1a and b). At diagnosis, mutations in CLL driver genes [29, 30] were found in 9 out of 12 (75%) patients (mean ± SEM of 1.4 ± 1.2 drivers per patient) (Fig. 1a). Among these, only one patient (CLL51) showed increased size of drivers CCF at progression: two variants in *NFKBIE* and *ATM* genes rised at progression (Fig. 1b, in bold red). Also in this patient, two additional variants from the same genes had fixed CCF between time points (Fig. 1b, in bold black). Furthermore, one patient (CLL31) among those without alterations in driver genes acquired a mutation at progression affecting the gene *TENM1* (CCF = 0.31) (Fig. 1b), not previously associated with CLL.

We also analyzed the mutational status of 9 CLL driver genes (*TP53, BIRC3, ATM, NOTCH1, SF3B1, XPO1, MYD88, FBXW7* and *POT1*) by targeted next generation sequencing in the same patients. With a mean read depth of 2000X, the limit of detection was set at a minimum coverage of 100 reads and a minimum of 0.05 VAF. Despite high depth sequencing, no additional changes over time affecting these drivers were observed (Supplementary Table S4).

Regarding changes in CNVs by WES, at diagnosis, CNVs were detected in 10 out of 12 (83%) patients with a mean ± SEM of 4.0 ± 4.1 (range 1–12) CNVs per patient (Supplementary Table S5 and Supplementary Fig. S1). Seven out of 12 (58%) patients had recurrent CNVs associated with CLL, including del(13q), tri(12), del(11q) and del(17p)(3), but all remained stable over time. Nonetheless, we did observe acquisition of del(8p) and del(15p) with a CCF of 72 and 44%, respectively, at progression in the same patient that showed increased CCF in driver genes (CLL51).

For patients without clinical progression, we analyzed the panel of CLL driver genes in paired B-CLL cells at diagnosis and asymptomatic follow-up (*n* = 9 patients). We identified mutations at diagnosis in 4 out of 9 (44%) non-progressing patients. One of them (CLL23) displayed increased VAF in one *ATM* variant*,* and another one (CLL47) showed reduced VAF in one mutation affecting *FBXW7* at second sampling (Supplementary Table S4).

Altogether, these results indicate that adquisition or expansion of genetic alterations is not linked to naturally occurring clinical progression, as previously reported by others [6–12, 14, 15].

### Effector memory CD8^+^ T cells co-expressing inhibitory receptors accumulate at CLL progression while they remain steady over time in patients with stable disease

Prior studies in patients diagnosed with CLL have shown an increase in defective circulating CD8^+^ T cells displaying a terminally differentiated phenotype compared to aged-matched healthy donors [18–21, 31, 32]. However, how these CD8^+^ T cells potentially evolve from diagnosis to clinical progression using longitudinal samples has not yet been studied. To investigate this, we analyzed the immunophenotype of T cells in paired PBMC samples from our two cohorts: 19 patients were analyzed at time of diagnosis and progression before treatment and 10 patients were analyzed at time of diagnosis and a second time point during follow-up without progression (from here referred to as “non-progression”). Firstly, the CD4/CD8 ratio at second sampling was significantly decreased over time only in patients that progressed, in whom effector memory CD8^+^ T cells (T_EM_: CCR7^−^CD45RA^−^) were the sole expanded T cell subset at progression, whereas no significant changes in CD8^+^ T cells maturation subsets were observed in non-progressors (Supplementary Fig. S2a and b).

**Fig. 2:**
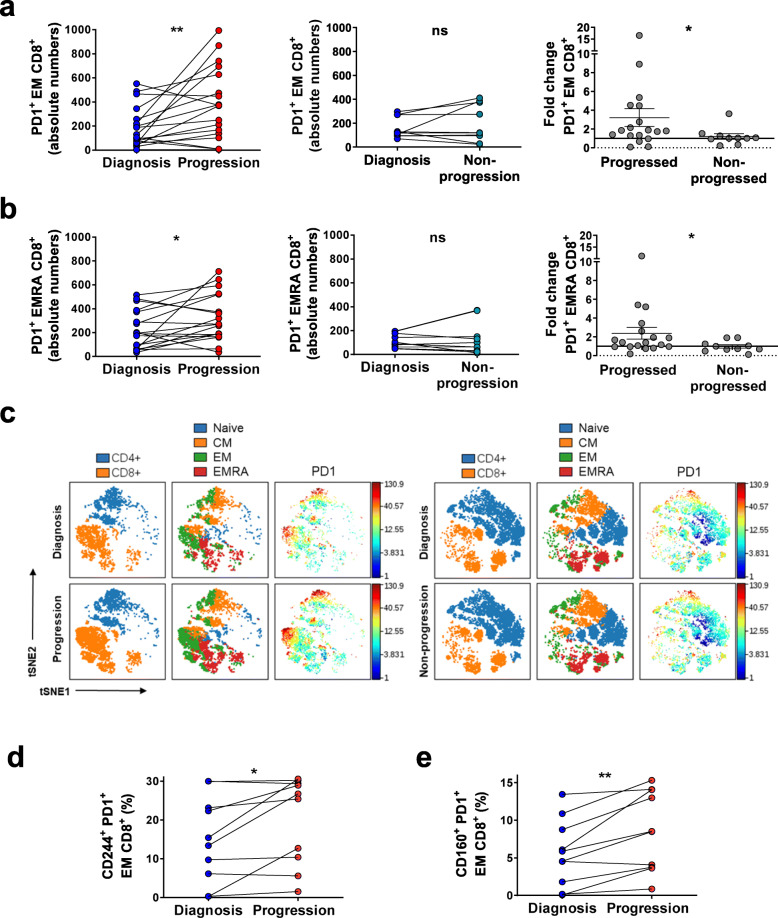
PD1^+^ effector memory CD8^+^ T cells subsets and co-expression of inhibitory receptors in progressing and non-progressing CLL patients. **a** Absolute numbers of PD1^+^ EM and **b** PD1^+^ EMRA CD8^+^ T cells in progressing (left, *n* = 18) and non-progressing patients (middle, *n* = 10) at diagnosis and progression or non-progression. Fold change between time points of PD1^+^ EM and PD1^+^ EMRA CD8^+^ cells in progressing and non-progressing patients (right). **c** Representative viSNE plots of T cell differentiation subsets (naïve: CCR7^+^CD45RA^+^; central memory, CM: CCR7^+^CD45RA^−^; effector memory, EM: CCR7^−^CD45RA^−^ and EM CD45RA^+^, EMRA: CCR7^−^CD45RA^+^) and PD1 expression in CD4^+^ and CD8^+^ T cells at the two time points from one representative patient from each group. **d** Percentages of EM CD8^+^ cells co-expressing CD244 and PD1 (*n* = 12) and **e** CD160 and PD1 (*n* = 10). Graphs show mean ± SEM or paired values. (*P < 0.05; **P < 0.01; ***P < 0.001; Wilcoxon matched paired test or Mann-Whitney test)

PD1 is expressed in chronically stimulated CD8^+^ T cells and has a relevant role in T cell exhaustion [33, 34]. It also has been described as preferentially expressed in effector memory CD8^+^ T cells, which are increased in CLL compared to healthy donors [18, 21]. Here, we observed an accumulation of PD1-expressing CD8^+^ T cells (Supplementary Fig. S2c) and an enrichment in PD1^+^ T_EM_ and PD1^+^ T_EMRA_ CD8^+^ subsets. This increase in exhausted CD8^+^ T cells was not observed at non-progression (Fig. 2a to c). To further investigate whether other features of T cell exhaustion and impaired effector function in CD8^+^ T cells were also enhanced at CLL progression, we measured the co-expression of PD1 with other inhibitory receptors, namely CD244 and CD160 [33, 35]. In line with our previous findings, we observed that CD8^+^ T cells co-expressing PD1 with CD244 or CD160 significantly rose only at progression, although the fold-change increase in CD8^+^ T cells expressing PD1^+^CD160^+^ was not significantly higher than the one observed in non-progressing patients (Supplementary Fig. S2d to f). In addition, the expanded T_EM_ CD8^+^ subset in progressing patients gained features of severe exhaustion at the time of progression as denoted by higher co-expression of PD1 with CD244 or CD160 (Fig. 2d and e). Altogether, we observed a longitudinal increase from diagnosis to progression in antigen-experienced effector memory CD8^+^ T cell subsets together with an increased co-expression of inhibitory receptors. On the contrary, no significant changes were observed over time in patients that did not progress.

### Terminally exhausted CD8^+^ T cells (T-bet^dim/−^Eomes^hi^PD1^hi^) accumulate at CLL progression

**Fig. 3:**
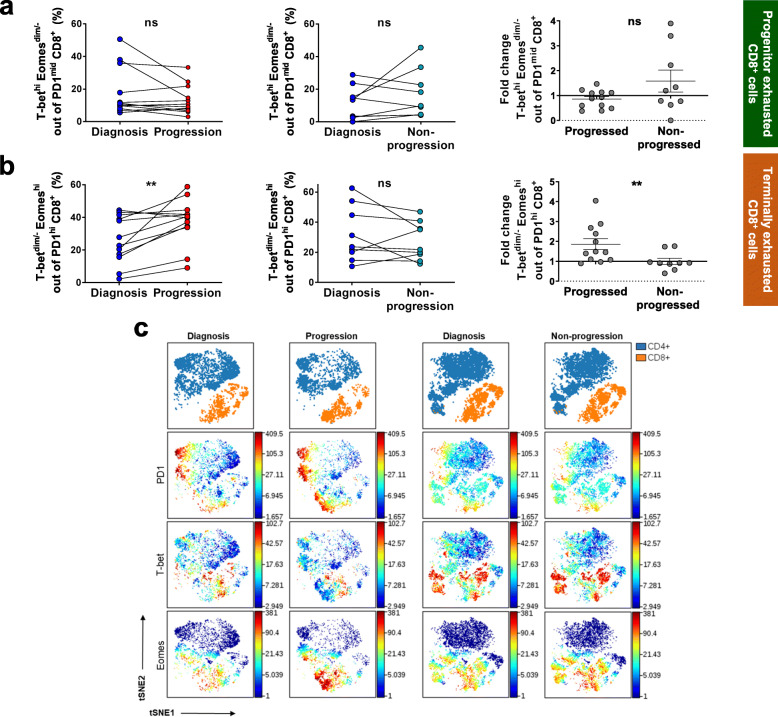
Progenitor (T-bet^hi^Eomes^dim/−^PD1^mid^) and terminally (T-bet^dim/−^Eomes^hi^PD1^hi^) exhausted CD8^+^ T cells in progressing and non-progressing CLL patients. **a** Percentages of T-bet^hi^Eomes^dim/−^ out of PD1^mid^CD8^+^ T cells and **b** T-bet^dim/−^Eomes^hi^ out of PD1^hi^CD8^+^ T cells in progressing (left, n = 12) and non-progressing patients (middle, *n* = 9) at diagnosis and progression or non-progression. Fold change between time points of both subsets comparing progressing and non-progressing patients (right). **c** Representative viSNE plots of PD1, T-bet and Eomes expression in CD4^+^ and CD8^+^ T cells at the two time points from one representative patient from each group. Graphs show mean ± SEM or paired values. (*P < 0.05; **P < 0.01; ***P < 0.001; Wilcoxon matched paired test or Mann-Whitney test)

T-bet and Eomesodermin (Eomes) are two T-box transcription factors that regulate the differentiation process of CD8^+^ T cells after antigen encounter and cooperate in the maintenance of long-term immunity [36]. Several studies have pointed out that they both have roles in CD8^+^ T cell exhaustion. T-bet represses PD1 expression and other inhibitory receptors [37] while Eomes, in contrast, is related to high expression of multiple inhibitory receptors, including PD1 [38, 39]. Accordingly, differential expression of both transcription factors combined with moderate or high PD1 levels defines two distinctly exhausted CD8^+^ T cell subsets: the progenitor (T-bet^hi^Eomes^dim/−^PD1^mid^) and the terminal progeny (T-bet^dim/−^Eomes^hi^PD1^hi^) [38] (Supplementary Fig. S3). Moreover, higher expression of Eomes in CD8^+^ T cells has been observed in patients diagnosed with CLL compared to HD and its function is essential for proper T cell expansion in CLL mouse models [40]. Since we observed that CD8^+^ T cells from CLL patients gained features of a more severe exhaustion degree at progression, we hypothesized that the terminal progeny would also be increased at progression. Indeed, we found that the CD8^+^ progenitor subset remained stable over time in both progressors and non-progressors (Fig. 3a and c), while the terminally exhausted CD8^+^ subpopulation (T-bet^dim/−^Eomes^hi^PD1^hi^) was significantly increased only in progressing patients (Fig. 3b and c). These findings confirm that CD8^+^ T cells at progression exhibit a severe terminal exhaustion condition likely impairing their ability to control the growth of malignant cells.

### T cells acquire a distinct transcriptional profile at CLL progression

**Fig. 4:**
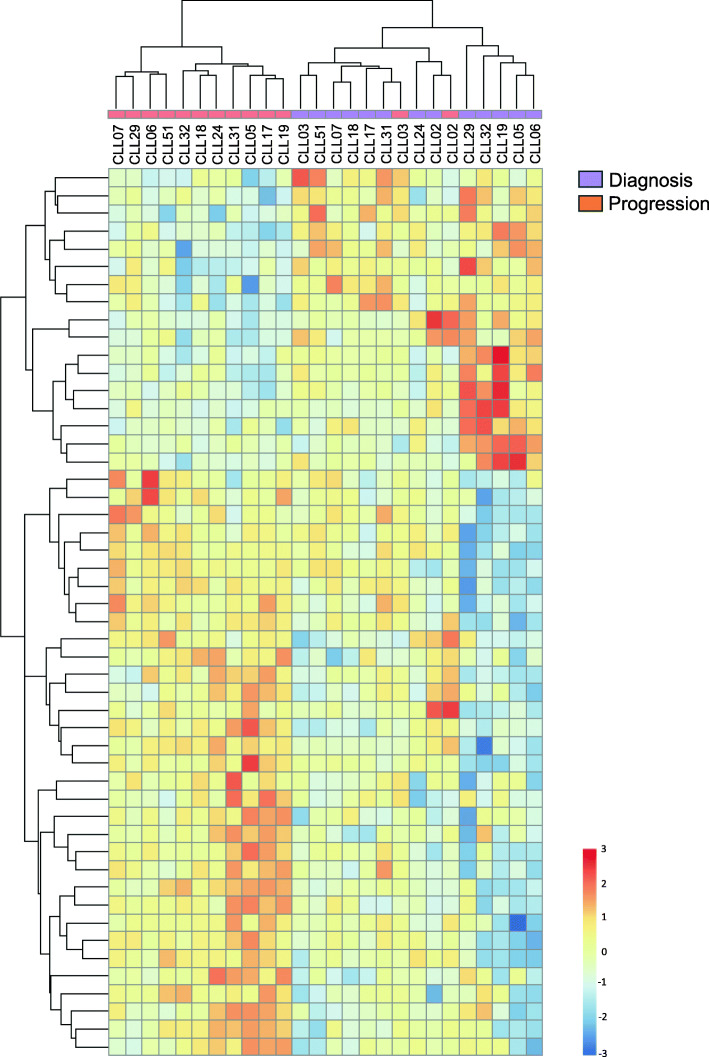
RNA-Seq of T cells from progressing CLL patients. Heatmap showing the unsupervised hierarchical clustering of the top-50 differentially expressed genes from paired sorted T cells at diagnosis and progression (*n* = 13)

In order to broadly characterize the alterations that occur over time in T cells from patients diagnosed with CLL cells related to clinical progression, we performed paired RNA-Seq analysis on isolated T cells (mean purity of 92%): 13 patients were analyzed at time of diagnosis and progression and 6 patients were analyzed at diagnosis and at follow-up (non-progression). After selecting uniquely mapped reads, the unsupervised hierarchical clustering analysis of paired samples from progressing patients defined two main clusters: one corresponding exclusively to T cells at progression and another cluster including T cells at diagnosis plus two samples at progression, highlighting that the transcriptional profile of T cells at progression was clearly distinct from that of T cells at diagnosis (Fig. 4). A total of 80 genes (including protein coding and lncRNA transcripts) were significantly up or downregulated in T cells from diagnosis to progression, while in contrast only 3 genes were differentially expressed in T cells from non-progressed patients at the time of follow-up after supervised analysis (all genes padj< 0.05 are detailed in Supplementary Table S6). Moreover, those 3 differentially expressed genes found in non-progressing patients were also found at progression. Briefly, the transcriptional profile of T cells at progression suggests lower mobility and differentiation capacity as well as an impairment in mitochondrial oxidative phosphorylation (Supplementary Table S7), essential processes for the maintenance of T cell effector functions [41]. Additionally, genes related to fatty acids and amino acids catabolism and glucose transporters were upregulated, while lower expression of genes related to the synthesis of cellular components and RNA processing mechanisms were identified at progression, suggesting a potentially dysregulated T cell metabolism. T cells at progression also showed upregulation of genes associated with immune response and exhaustion [42–44]. Collectively, these results point towards an impaired cytoskeleton formation, mitochondrial metabolism and immune dysregulation, consistent with the exhausted and dysfunctional status of T cells that is aggravated at CLL progression.

### CLL cells from patients at clinical progression have enhanced capacity to induce PD1 expression in CD8^+^ T cells via soluble factors including IL-10

**Fig. 5:**
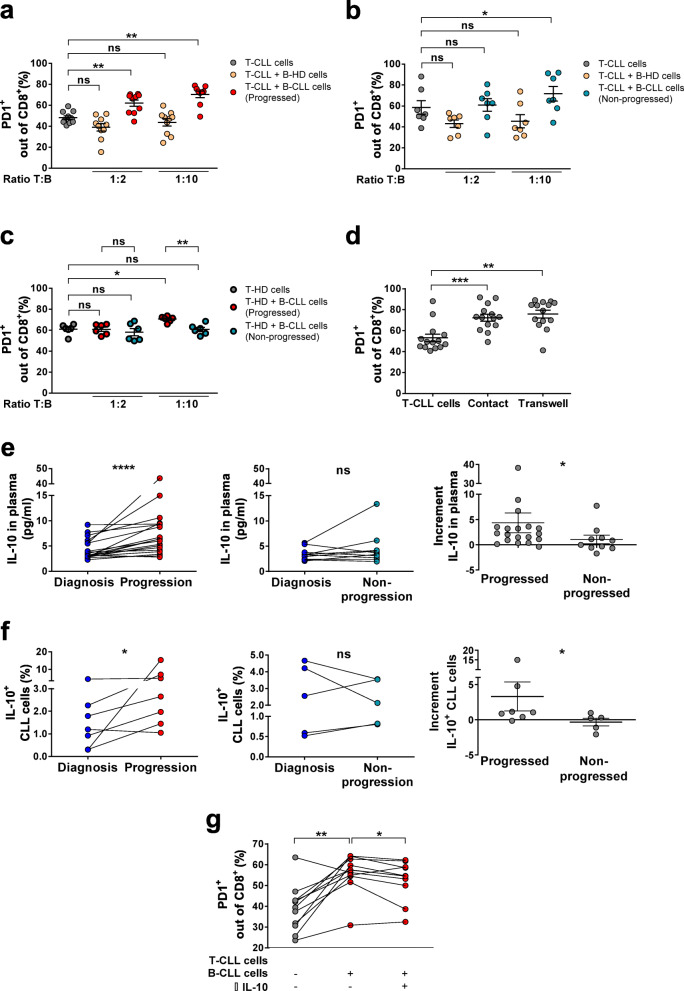
PD1 expression in CD8^+^ T cells after co-culture with B-HD cells or B-CLL cells and contribution of soluble factors to PD1 expression. **a** Percentages of PD1^+^ CD8^+^ T cells from progressing CLL patients after stimulation with anti-CD3 and anti-CD28 for 7 days (grey dots) and in presence of B-HD cells (yellow dots) or B-CLL cells at the time of progression (red dots) at the indicated T:B ratios (n = 10). **b** Percentages of PD1^+^CD8^+^ T cells from non-progressing CLL patients after stimulation with anti-CD3 and anti-CD28 for 7 days (grey dots) and in presence of B-HD cells (yellow dots) or B-CLL cells at asymptomatic follow-up (blue dots) at the indicated T:B ratios (*n* = 7). **c** Percentages of PD1^+^CD8^+^ T cells from age-matched healthy donors (T-HD) after stimulation with anti-CD3 and anti-CD28 for 7 days (grey dots) and in presence of B-CLL cells at progression (bold red dots) or B-CLL cells at asymptomatic follow-up (bold blue dots) at the indicated T:B ratios (*n* = 6). **d** Percentages of PD1^+^CD8^+^ T cells from CLL patients after stimulation with anti-CD3 and anti-CD28 for 7 days and in contact with B-CLL cells or separated by transwell inserts at 1:10 T:B ratio (*n* = 14). **e** Concentration (pg/ml) of IL-10 in plasma from progressing (left, *n* = 19) and non-progressing patients (middle, n = 10). Increment of IL-10 in plasma between time points comparing progressing and non-progressing patients (right). **f** Percentage of IL-10^+^ CLL cells in progressing (left, n = 7) and non-progressing patients (middle, *n* = 5) at the two time points after co-culture of paired PBMCs with bone marrow stromal UE6E7T-2 cells, CD40L and TLR9L for 48 h. Increment of IL-10^+^ CLL cells between time points in progressing and non-progressing patients (right). **g** Percentage of PD1^+^CD8^+^ T cells from CLL after 7-day co-culture with progressing B-CLL cells at 1:10 T:B ratio, following the protocol previously described, and after adding anti-human IL-10 neutralizing antibody (*n* = 11). Graphs show mean ± SEM or paired values (*P < 0.05; **P < 0.01; ***P < 0.001; ****P < 0.0001; Wilcoxon matched paired test or Mann-Whitney test)

The observed increased exhaustion at progression compared to patients that had not progressed could be mainly caused by a potentially increased interaction with malignant cells due to higher tumoral load; alternatively, malignant cells at progression could be intrinsically more capable of inducing exhaustion in T cells. In order to gain insight into the functional mechanisms that could trigger said increased exhaustion of CD8^+^ T cells, we compared the effect on T cells of malignant B-CLL cells obtained from patients at clinical progression vs. malignant cells from patients at follow-up of stable disease (non-progression). To do that we firstly co-cultured T cells from patients with CLL (T-CLL) with increasing concentrations of either healthy B cells (B-HD) or autologous B-CLL cells obtained from patients at progression or from patients at follow-up (non-progression). After 7 days of co-culture, we found that PD1 expression was increased in CD8^+^ T cells in presence of progressed B-CLL cells at any leukemic cell to T cell ratio, while B-HD cells did not induce changes in PD1 expression (Fig. 5a). Moreover, B-CLL cells from patients that did not progress were only able to induce PD1 expression in autologous CD8^+^ T cells at the highest B to T cell ratio tested, suggesting that there are intrinsic characteristics in malignant B cells from patients that are in need of treatment that can contribute to the observed T cell exhaustion. The same results were obtained when looking at PD1 and CD244 co-expression (supplementary Fig. S4a). Therefore, and in order to further compare the capacity to induce exhaustion of malignant B cells from patients at progression versus malignant cells from non-progressing patients, we co-cultured T cells from healthy donors (T-HD) with both types of malignant cells. In this setting, only progressed B-CLL cells were capable of inducing PD1 expression in CD8^+^ T cells whereas non-progressed B-CLL cells were not (Fig. 5c), while they showed no differences in the induction of PD1 and CD244 co-expression. Thus, although we cannot completely rule out that the observed enhanced exhaustion at progression is merely caused by increased tumoral load, these results indicate that malignant cells at progression are intrinsically more capable of inducing PD-1 expression in both autologous or HD-derived T cells.

To investigate whether this T cell state induced by CLL cells occurs through a cell-to-cell mediated mechanism or, otherwise, is mediated by soluble factors, T-CLL and B-CLL co-cultures were performed with transwells. We observed that the induction of PD1 and CD244 in CD8^+^ T cells was equivalent when there was no contact between autologous T cells and leukemic cells (Fig. 5d and Supplementary Fig. S4c, suggesting that secretion of soluble factors lead to upregulation of exhaustion markers. In this regard, CLL cells are known to exhibit features of regulatory B cells, such as IL-10 production, [24] which has been found to have immunosuppressive function in CLL [45, 46], be related to shorter overall survival [47] and to correlate with the expression of PD1 in tumor infiltrating lymphocytes in solid tumors [48]. Based on this, we initially measured the levels of IL-10 in paired plasma samples from CLL patients and found that plasmatic IL-10 significantly increased at progression while remained stable over time in non-progressing patients (Fig. 5e). Next, to investigate whether malignant cells acquire an increased capacity to produce IL-10 at progression and, therefore, higher plasmatic levels are not simply reflecting the higher tumoral load at progression, we assessed the production of IL-10 by CLL cells in vitro after microenvironmental stimuli [27]. We detected an increased percentage of IL-10^+^ CLL cells only in malignant cells obtained at progression (Fig. 5f and Supplementary Fig. S4d), indicating that leukemic cells from patients at clinical progression have indeed increased potential to create an immunosuppressive microenvironment. Moreover, the induction of PD1 expression in vitro in CD8^+^ T cells was partially blocked after IL-10 neutralization (Fig. 5g). Finally, we analyzed the potential accumulation of MDSCs as an additional source of plasmatic IL-10, and we observed [49] an accumulation of these immunosupresive cells over time in all CLL patients regardless of their clinical evolution, although the degree of increment was higher in progressing patients (Supplementary Fig. S4e). These observations suggest that at the time of progression not only there is an increased load of leukemic cells, but these also have a higher IL-10 capacity production, which can contribute to the increased induction of CD8^+^ T cell exhaustion.

## Discussion

The biological processes that lead to clinical progression from early asymptomatic stages in patients diagnosed with CLL are not well understood and, consequently, the pathogenesis behind the natural history of this disease remains unclear. In this regard, longitudinal studies from diagnosis to clinical progression are essential to elucidate these mechanisms. To the best of our knowledge, this is the first comprehensive longitudinal analysis of the genetic and immunological processes driving disease progression in CLL. The longitudinal molecular study herein presented showed limited and non-recurrent molecular changes in CLL cells at progression, indicating that genetic clonal evolution is not the main driver of clinical progression, like previously observed in other series [6–15]. Conversely, longitudinal studies focused on potential changes in the immune microenvironment have not yet been conducted. Our analysis on the immune kinetics found an increasingly dysfunctional CD8^+^ T cell compartment in progressing patients that was not observed in those patients that remained asymptomatic. Moreover, we showed that soluble factors, such as IL-10 produced by CLL cells, play a role in CD8^+^ T cell exhaustion and in the progression of the disease.

An altered anti-tumor immune response is evidenced in CLL by diverse factors affecting mainly T cells [18–21, 31, 32]. Recent studies using the Eμ-TCL1 mouse model indicate that CD8^+^ T cells can delay CLL progression at the same time that their expression of inhibitory receptors progressively increases [50]. Also, although only studied in 3 cases, in spontaneously regressing CLL the expression of PD1 in CD8^+^ T cells also decreased when malignant cells declined [51]. Accordingly, our longitudinal immune analysis in CLL patients showed that effector memory CD8^+^ T cell subsets expressing PD1 accumulated specifically at clinical progression. In addition, EM CD8^+^ T cells co-expressing PD1 with CD244 and CD160 and terminally exhausted (T-bet^dim/−^Eomes^hi^PD1^hi^) CD8^+^ T cells were significantly increased at progression, denoting severe dysfunction in CD8^+^ T cells over time. Importantly, these changes were not observed in non-progressing patients, supporting the primary role of immune dysfunction in CLL progression.

Broad analysis of transcriptomic changes also revealed significantly different expression profiles in T cells at progression compared to diagnosis while only minor differences were found in non-progressing patients along time. Although the low quality of the RNA we obtained hampered a broader analysis, we were able to identify changes in the transcriptome specifically associated with clinical progression when using only uniquely mapped reads to avoid potential artifacts due to low input.

In this study, we also identified that CLL cells obtained at the time of progression had enhanced capacity of inducing exhaustion in both autologous and HD-derived CD8^+^ T cells compared to malignant CLL cells from patients with stable disease, as well as increased capacity to secrete IL-10 upon microenvironmental stimuli. In addition, we found that PD1 upregulation depended mainly on soluble factors. Therefore, although the accumulation of exhausted CD8^+^ T cells in progressing patients might be merely the consequence of the higher tumoral load characterizing progression in the majority of patients (see Supplementary Table S2 for clinical data), our data showing increased in vitro capacity to induce T cell exhaustion and to produce IL-10 are compatible with an scenario where accumulating malignant cells also acquire higher immunosuppressive properties along the course of the disease only in patients that will eventually progress. These cells would then promote engagement in a positive feed-back system further increasing CD8^+^ T cell exhaustion and ultimately facilitating the transition to clinical progression. In this regard, Gonnord et al. recently described that CD8^+^ T cells from untreated CLL patients that will need therapy within 6 months after analysis display an unique signature which is not correlated with the time that CD8^+^ T cells have been exposed to CLL cells [52], indicating again that the exhaustion of T cells is not a mere product of increased exposure to malignant cells, either in time or in tumoral load. Further studies trying to definitively figure out the mechanisms behind the coevolution of the immune system and malignant cells in CLL are definitively worth performing.

Our results provide pre-clinical evidence to support the design of clinical studies aimed at improving anti-tumoral T cell responses. This may be particularly advantageous in the context of treatment with autologous chimeric antigen receptor (CAR)- T cells derived from patients in an advanced disease stage, which display low cytotoxic activity and achieve low response rates [53]. Notably, immunomodulatory effects of ibrutinib therapy are able to recover T cell function and subsequently increase the response rate to CAR-T cell therapy [54, 55]. Moreover, these results lay the foundations for the clinical testing of immunotherapy in early stages of the disease to prevent or delay clinical progression. In addition, the identification of signs of increased immunosuppression could be exploited in the future to improve early detection of patients that will likely progress.

## Conclusions

Collectively, our findings indicate that at clinical progression CLL cells exhibit limited genetic changes from diagnosis, while CD8^+^ T cells show increased exhaustion that can be induced by IL-10 secreted by malignant B cells. In contrast, patients that remain stable over time did not experience significant changes in their genetic or T cell compartments. Also, malignant cells from patients at clinical progression showed enhanced capacity to induce exhaustion-related features in CD8^+^ T cells ex vivo.

These results provide evidence to support the exploration of immunotherapeutic interventions in early stages aiming at avoiding or delaying clinical progression of the disease. Additionally, further analysis of both immunological and malignant B-CLL cell-intrinsic factors in larger cohorts of patients will help us better identify those patients that are more likely to progress shortly after diagnosis.

## Supplementary Information

**Additional file 1: Supplementary Table S2.** Detailed clinical characteristics from CLL patients. **Supplementary Table S3.** SNVs and Indels analysis by WES in progressing CLL patients. **Supplementary Table S4.** CLL driver gene analysis (*TP53, BIRC3, ATM, NOTCH1, SF3B1, XPO1, MYD88, FBXW7* and *POT1*) by NGS in progressing and non-progressing CLL patients. **Supplementary Table S5.** CNVs analysis by WES in progressing CLL patients. **Supplementary Table S6.** Differentially expressed genes in T cells from CLL patients.

**Additional file 2: Supplementary Table S1.** Monoclonal antibodies (mAbs). **Supplementary Table S7.** Highlighted dysregulated genes in T CLL cells at progression. **Supplementary Figure S1.** Longitudinal analysis of the CCF of CNVs from paired B-CLL cells at diagnosis and progression before treatment. Comparison of the CCF with 95% CI for each CNV detected per patient (*n* = 10) between diagnosis and progression. Significantly increased (red lines) and stable CCF (grey lines) are shown. Recurrent CNVs in CLL (del(13q), del(11q), del(17p) and +[12]) are plotted with bold lines and labeled with CNV name: stable CCF (bold black) is shown. **Supplementary Figure S2.** CD8^+^ T cell differentiation subsets and PD1 expression in CD8^+^ T cells from progressing and non-progressing CLL patients. **a** CD4/CD8 ratio in progressing (*n* = 19) and non-progressing patients (*n* = 10) at diagnosis and progression or non-progression. **b** Absolute numbers of CD8^+^ T cell differentiation subsets (naive: CCR7^+^CD45RA^+^; central memory, CM: CCR7^+^CD45RA^−^; effector memory, EM: CCR7^−^CD45RA^−^ and EM CD45RA^+^, EMRA: CCR7^−^CD45RA^+^) in progressing (*n* = 19) and non-progressing patients (*n* = 10) at diagnosis and progression or non-progression. **c** Absolute numbers of PD1^+^CD8^+^ T cells in progressing (left, *n* = 19) and non-progressing patients (middle, *n* = 10) at diagnosis and progression or non-progression. Fold change of PD1^+^CD8^+^ T cells between time points comparing progressing and non-progressing patients (right). **d** Percentage of PD1^+^CD244^+^ CD8^+^ T cells in progressing (left, *n* = 12) and non-progressing patients (middle, *n* = 9) at diagnosis and progression or non-progression. Fold change of PD1^+^CD244^+^CD8^+^ T cells between time points comparing progressing and non-progressing patients (right). **e** Percentage of PD1^+^ 160^+^ CD8^+^ T cells in progressing (left, *n* = 12) and non-progressing patients (middle, *n* = 9) at diagnosis and progression or non-progression. Fold change of PD1^+^CD160^+^CD8^+^ T cells between time points comparing progressing and non-progressing patients (right). **f** Density plots of PD1, CD160 and CD244 coexpression in CD8^+^ T cells in representative patients at diagnosis and progression and at diagnosis and non-progression. Graphs show mean ± SEM or paired values (*P < 0.05; **P < 0.01; ***P < 0.001; Wilcoxon matched paired test or Mann-Whitney test). **Supplementary Figure S3.** Flow cytometric analysis of progenitor and terminal CD8^+^ subsets. Gating strategy followed for the identification of T-bet^hi^Eomes^dim/−^PD1^mid^ and T-bet^dim/−^Eomes^hi^PD1^hi^ CD8^+^ populations. **Supplementary Figure S4.** Co-expression of PD1 and CD244 in CD8^+^ T cells after co-culture with B-CLL cells. MDSCs in progressing and non-progressing CLL patients. **a** Percentages of PD1^+^CD244^+^ cells out of CD8^+^ T cells from progressing (left) and non-progressing (right) CLL patients after stimulation with anti-CD3 and anti-CD28 for 7 days (grey dots) and in presence of B-HD cells (yellow dots) or B-CLL cells at the time of progression (red dots, *n* = 10) or asymptomatic follow-up (blue dots, *n* = 7) at the indicated T:B ratios. **b** Percentages of PD1^+^CD244^+^ cells out of CD8^+^ T cells from healthy age-matched donors (T-HD) after stimulation with anti-CD3 and anti-CD28 for 7 days (grey dots) and in presence of B-CLL cells at progression (bold red dots) or B-CLL cells at asymptomatic follow-up (bold blue dots) at the indicated T:B ratios. **c** Percentages of CD8^+^ T cells from CLL patients co-expressing PD1 and CD244 after stimulation with anti-CD3 and anti-CD28 for 7 days and in contact with B-CLL cells or separated by transwell inserts at 1:10 T:B ratio for 7 days (*n* = 14).**d** Dot plots of IL-10^+^ B cells gated on CD19^+^CD5^+^ cells after 5 h of leukocyte stimulation (PIB), or brefeldin A (BFA) as control, from one representative patient from the progressed and non-progressed groups respectively. **e** Percentage of MDSCs (CD14^+^HLA-DR^low/−^) out of CD14^+^ cells in progressing (left, *n* = 17) and non-progressing patients (middle, *n* = 10) at diagnosis and progression or non-progression. Increment of MDSCs between time points comparing progressing and non-progressing patients (right). Graphs show mean ± SEM or paired values (*P < 0.05; **P < 0.01; ***P < 0.001; ****P < 0.0001; Wilcoxon matched paired test or Mann-Whitney test).

## Data Availability

For original data, please contact the corresponding author. WES and RNA-Seq data generated during the current study are deposited at EGA and GEO under accession numbers EGAS00001004116 and GSE141787, respectively.

## Abbreviations

CAR: chimeric antigen receptor
CCF: cancer cell fraction
CI: confidence interval
CLL: chronic lymphocytic leukemia
CNV: copy number variation
IWCLL: international workshop on CLL
MDSC: myeloid-derived suppressor cells
PBMCs: peripheral blood mononuclear cells
RNA-seq: RNA sequencing
SEM: standard error of the mean
TTP: time to progression
VAF: variant allele fraction
WES: whole exome sequencing
