# Supplementary material for: Immunological and genetic kinetics from diagnosis to clinical progression in chronic lymphocytic leukemia
Source: Biomark Res. 2021 May 20;9:37. doi: 10.1186/s40364-021-00290-z (PMC8138982; doi:10.1186/s40364-021-00290-z)
Supplement: Supplementary file 2 — Additional file 2: Supplementary Table S1. Monoclonal antibodies (mAbs). Supplementary Table S7. Highlighted dysregulated genes in T CLL cells at progression. Supplementary Figure S1. Longitudinal analysis of the CCF of CNVs from paired B-CLL cells at diagnosis and progression before treatment. Comparison of the CCF with 95% CI for each CNV detected per patient (n = 10) between diagnosis and progression. Significantly increased (red lines) and stable CCF (grey lines) are shown. Recurrent CNVs in CLL (del(13q), del(11q), del(17p) and +[12]) are plotted with bold lines and labeled with CNV name: stable CCF (bold black) is shown. Supplementary Figure S2. CD8+ T cell differentiation subsets and PD1 expression in CD8+ T cells from progressing and non-progressing CLL patients. a CD4/CD8 ratio in progressing (n = 19) and non-progressing patients (n = 10) at diagnosis and progression or non-progression. b Absolute numbers of CD8+ T cell differentiation subsets (naive: CCR7+CD45RA+; central memory, CM: CCR7+CD45RA−; effector memory, EM: CCR7−CD45RA− and EM CD45RA+, EMRA: CCR7−CD45RA+) in progressing (n = 19) and non-progressing patients (n = 10) at diagnosis and progression or non-progression. c Absolute numbers of PD1+CD8+ T cells in progressing (left, n = 19) and non-progressing patients (middle, n = 10) at diagnosis and progression or non-progression. Fold change of PD1+CD8+ T cells between time points comparing progressing and non-progressing patients (right). d Percentage of PD1+CD244+ CD8+ T cells in progressing (left, n = 12) and non-progressing patients (middle, n = 9) at diagnosis and progression or non-progression. Fold change of PD1+CD244+CD8+ T cells between time points comparing progressing and non-progressing patients (right). e Percentage of PD1+ 160+ CD8+ T cells in progressing (left, n = 12) and non-progressing patients (middle, n = 9) at diagnosis and progression or non-progression. Fold change of PD1+CD160+CD8+ T cells between time points comparing progres [file 40364_2021_290_MOESM2_ESM.docx]

**SUPPLEMENTARY INFORMATION**

**SUPPLEMENTARY METHODS**

**Primary samples**

PBMCs were isolated from whole blood by Ficoll-Paque Plus (GE Healthcare, Chicago, IL, USA) density gradient and cryopreserved in RPMI-1640 medium (Biowest, Nuaillé, France) with 10% dimethyl sulfoxide (DMSO, Sigma-Aldrich, St. Louis, MO, USA) and 10% heat-inactivated fetal bovine serum (FBS, Gibco^TM^, ThermoFisher Scientific, Waltham, MA, USA). Simultaneously, granulocytes were isolated by sedimentation with 2% dextran (Sigma-Aldrich).

**Isolation of B and T lymphocytes**

B-CLL and T-CLL cells were immunomagnetically isolated using the EasySep^TM^ Human B cell Enrichment Kit without CD43 Depletion and the EasySep^TM^ Human T cell Isolation Kit (StemCell, Vancouver, Canada) respectively. The purity of isolated cells was >90% CD19^+^CD5^+^ and >85% CD3^+^ as assessed by flow cytometry.

**DNA and RNA preparation for WES and RNA-Seq**

Genomic DNA was extracted from isolated B-CLL cells and T cells or granulocytes as germline controls using the AllPrep DNA/RNA (Qiagen, Hilden, Germany) Kit. RNA was also extracted from isolated T-CLL cells.

**WES and data processing**

*Sample preparation and sequencing:* 200ng of tumor or germline (T cells or granulocytes) DNA were used for SureSelect Human All Exon V5 (Agilent Technologies, Santa Clara, CA, USA) whole exome capture-based library preparation. Genomic DNA was sheared on a Covaris E210 and purified/size selected with AMPure XP beads (Beckman Coulter, Brea, CA, USA). The sheared DNA was end-repaired, 3´ adenylated and ligated to NGS sequencing adapters. The adapter-modified DNA was amplified pre-capture through 10 PCR cycles. The PCR product was quality controlled on the Agilent 2100 Bioanalyzer 7500 chip (Agilent Technologies) to confirm size range (200 to 350bp) and quantity and hybridized for 24h at 65°C. The hybridization mix was washed and the eluate was post-capture PCR amplified (12 cycles) in order to add the index tags. The final library size and concentration were determined on Agilent 2100 Bioanalyzer 7500 chip. Libraries were sequenced on HiSeq2500 (Illumina, San Diego, CA, USA) using TruSeq SBS Kit v4 (Illumina), following the manufacturer's instructions. Each sample was sequenced multiple times to achieve 110x mean depth of coverage.

*Data analysis:* raw FASTQ files were evaluated using quality control checks from FastQC (https://www.bioinformatics.babraham.ac.uk/projects/fastqc/) and Trimmomatic (1) was employed to remove low quality bases, adapters and other technical sequences. Then, alignment to the human reference genome (GRCh37/hg19) was done using BWA-mem (2), generating sorted BAM files with SAMtools (3). Optical and PCR duplicates were removed using Sambamba (4). SNVs and indels were identified using a variation of Sidrón algorithm, as previously described (5). Indels realignment was performed to correct underestimated allele frequencies. Finally, all variants were annotated with functional, population and cancer-related information.

*Variant calling and annotation*: indels were identified as previously described (5), with the following parameters: total read depth ≥6, mutated allele count ≥3, variant frequency ≥0.01, base quality ≥10, and mapping quality ≥20. Variants were annotated using several databases containing functional (Ensembl, CCDS, RefSeq, Pfam), population (dbSNP, 1000 Genomes, ESP6500, ExAC, gnomAD) and cancer-related (COSMIC – Release 87, ICGC – Release 27) information; as well as 14 scores from algorithms for prediction of the impact caused by variants on the protein structure and function (SIFT, SIFT 4G (6), PROVEAN (7), Mutation Assessor (8), Mutation Taster (9), LRT (10), MetaLR, MetaSVM (11), FATHMM, FATHMM-MKL, FATHMM-XF (12), primateAI (13) and Deogen2 (14)), and one score for evolutionary conservation of the affected nucleotide (GERP++) (15).

*Variant filtering:* variants with high frequency in the population (>0.01) were discarded. A minimum coverage of 20 reads and a minimum VAF of 0.1 in at least one time point were also established. Somatic status of each variant was defined using the Fisher Exact Test to compare tumor and germline control samples (p-value<0.01 and effect Size≥2.5). Only variants with a consistent damaging impact on protein were considered.

*Copy number variants (CNVs):* the exome2cnv algorithm used for CNVs detection incorporated a combination of read depth and allelic imbalance computations for copy number assessment.

*Cancer cell fraction (CCF):* the CCF and the 95% CI for each variant were calculated using the purity of samples determined by flow cytometry, the ploidy based on the copy number and the variant allele frequency. A significant change in CCF over time was determined if the 95% CIs of the CCF in the diagnosis and progression sample did not overlap (16).

**Targeted sequencing of CLL genetic drivers**

Sequencing of 9 CLL driver genes (*TP53*, *BIRC3*, *ATM*, *NOTCH1*, *SF3B1*, *XPO1*, *MYD88*, *FBXW7* and *POT1*) was performed using amplicon-based library preparation (CLL MASTR Plus assay; Multiplicom, Agilent) starting from 200ng of tumor DNA. Libraries were sequenced on HiSeq2500 (Illumina) with a read length of 250bp paired-end, achieving 2 000x mean depth of coverage. Limit of detection was set at VAF of 0.05 in at least one time point. Data analysis was performed using DNAnexus (DNAnexus, Mountain View, CA, USA).

**RNA-Seq and data processing**

*Sample preparation and sequencing:* 10ng of full-length T-cell-RNA were used to prepare sequencing libraries using the SMARTer Stranded Total RNA-Seq Kit v2 - Pico Input Mammalian (Takara, Kusatsu, Japan). Total T-cell-RNA was reverse transcribed and Illumina compatible adapters and indexes were added to the cDNA followed by a purification using Agencourt Ampure XP beads (Beckman Coulter). Next, ribosomal (18S and 28S) and mitochondrial (m12S and m16) cDNA transcripts were depleted and final libraries were amplified during 16 PCR cycles. After two consecutive purification steps, the product size distribution and the quantity were assessed using Bioanalyzer High Sensitivity DNA Kit (Agilent Technologies). Libraries were sequenced on HiSeq2500 (Illumina) using TruSeq SBS Kit v4 (Illumina). On average, 50 M paired-end reads were obtained per sample and 90% mapped to the reference genome.

*Data analysis:* reads were mapped against the human reference genome (GENCODE release 28) using STAR version 2.5.3a (17) with the parameter outFilterMultimapNmax=1 in order to ensure that only transcripts that were uniquely mapped to the human genome were analyzed so that potential artifacts can be avoided. Genes were quantified with RSEM version 1.3.0 (18) using the GENCODE release 28 human annotation. Differential expression analysis was performed adjusting for patient with DESeq2 version 1.18.1 (19). Genes with adjusted P value (padj)<0.05 were considered significant and filtered out if padj>0.05 and |shrunken fold change|<1.5. Heatmap showing the top-50 differentially expressed genes was performed with the regularized log transformation of the counts using the pheatmap R package with the option scale=”row”.

**Cell lines**

The UE6E7T-2 human bone marrow stromal cells (BMSCs) cell line was obtained from Riken Cell Bank (Ibakari, Japan) and authenticated using short tandem repeat analysis. Cells were cultured at 37ºC in 5% CO_2_ atmosphere in Dulbecco’s Modified Eagle Medium (DMEM; Biowest) supplemented with 10% FBS, 2mM L-glutamine and 50μg/mL penicillin/streptomycin (Biowest).

**Flow cytometry and cell staining**

For immunophenotypic analysis, cryopreserved PBMCs were thawed in RPMI-1640 supplemented with 10% FBS, 2mM L-glutamine and 50μg/mL penicillin/streptomycin, washed and stained with surface mAbs for 15 minutes at room temperature. Then, cells were resuspended in staining buffer (PBS with 1% bovine serum albumin and 0.1% sodium azide (Sigma-Aldrich)) and acquired in the flow cytometer.

For the staining of transcription factors and intracellular cytokines, cells were permeabilized for 30 minutes at 4ºC using the Foxp3/Transcription Factor Staining Buffer Set (eBioscience, San Diego, CA, USA) and incubated with mAbs for 30 minutes at room temperature.

Compensation was performed with single-stained tubes with VersaComp Antibody Capture beads (Beckman Coulter). The gating strategy used included only singlets and forward and side scatter live cells. All gates were based on fluorescence minus one (FMO) or isotype controls.

**B and T lymphocytes co-cultures**

B and T lymphocytes co-cultures were maintained in AIM V^TM^ Medium (Gibco^TM^, ThermoFisher Scientific) supplemented with 2% human plasma and 50μM β-mercaptoethanol (Gibco^TM^, ThermoFisher Scientific). Cells were stimulated with 1μg/mL anti-CD3 (Clone OKT3; Miltenyi Biotec, Bergisch Gladbach, Germany) and 1μg/mL anti-CD28 (Clone 15E8, Miltenyi Biotec). When indicated, 10μg/ml LEAF^TM^ purified anti-human IL-10 (BioLegend, San Diego, CA, USA) was added. After 7 days, cells were analyzed by flow cytometry. Assays were also performed using HTS Transwell-96 well plates (pore size 0.4μm; Corning, NY, USA).

**SUPPLEMENTARY REFERENCES**

1. Bolger AM, Lohse M, Usadel B. Trimmomatic: a flexible trimmer for Illumina sequence data. Bioinformatics. 2014;30:2114–20.

2. Li H, Durbin R. Fast and accurate long-read alignment with Burrows-Wheeler transform. Bioinformatics. 2010;26:589–95.

3. Li H, Handsaker B, Wysoker A, Fennell T, Ruan J, Homer N, et al. The Sequence Alignment/Map format and SAMtools. Bioinformatics. 2009;25:2078–9.

4. Tarasov A, Vilella AJ, Cuppen E, Nijman IJ, Prins P. Sambamba: fast processing of NGS alignment formats. Bioinformatics. 2015;31:2032–4.

5. Puente XS, Pinyol M, Quesada V, Conde L, Ordóñez GR, Villamor N, et al. Whole-genome sequencing identifies recurrent mutations in chronic lymphocytic leukaemia. Nature. 2011;475:101–5.

6. Kumar P, Henikoff S, Ng PC. Predicting the effects of coding non-synonymous variants on protein function using the SIFT algorithm. Nat Protoc. 2009;4:1073–81.

7. Choi Y, Sims GE, Murphy S, Miller JR, Chan AP. Predicting the functional effect of amino acid substitutions and indels. PLoS ONE. 2012;7:e46688.

8. Reva B, Antipin Y, Sander C. Predicting the functional impact of protein mutations: application to cancer genomics. Nucleic Acids Res. 2011;39:e118.

9. Schwarz JM, Cooper DN, Schuelke M, Seelow D. MutationTaster2: mutation prediction for the deep-sequencing age. Nat Methods. 2014;11:361–2.

10. Chun S, Fay JC. Identification of deleterious mutations within three human genomes. Genome Res. 2009;19:1553–61.

11. Dong C, Wei P, Jian X, Gibbs R, Boerwinkle E, Wang K, et al. Comparison and integration of deleteriousness prediction methods for nonsynonymous SNVs in whole exome sequencing studies. Hum Mol Genet. 2015;24:2125–37.

12. Shihab HA, Gough J, Mort M, Cooper DN, Day INM, Gaunt TR. Ranking non-synonymous single nucleotide polymorphisms based on disease concepts. Hum Genomics. 2014;8:11.

13. Sundaram L, Gao H, Padigepati SR, McRae JF, Li Y, Kosmicki JA, et al. Predicting the clinical impact of human mutation with deep neural networks. Nat Genet. 2018;50:1161–70.

14. Raimondi D, Tanyalcin I, Ferté J, Gazzo A, Orlando G, Lenaerts T, et al. DEOGEN2: prediction and interactive visualization of single amino acid variant deleteriousness in human proteins. Nucleic Acids Res. 2017;45:W201–6.

15. Davydov EV, Goode DL, Sirota M, Cooper GM, Sidow A, Batzoglou S. Identifying a high fraction of the human genome to be under selective constraint using GERP++. PLoS Comput Biol. 2010;6:e1001025.

16. Landau DA, Tausch E, Taylor-Weiner AN, Stewart C, Reiter JG, Bahlo J, et al. Mutations driving CLL and their evolution in progression and relapse. Nature. 2015;526:525–30.

17. Dobin A, Davis CA, Schlesinger F, Drenkow J, Zaleski C, Jha S, et al. STAR: ultrafast universal RNA-seq aligner. Bioinformatics. 2013;29:15–21.

18. Li B, Dewey CN. RSEM: accurate transcript quantification from RNA-Seq data with or without a reference genome. BMC Bioinformatics. 2011;12:323.

19. Love MI, Huber W, Anders S. Moderated estimation of fold change and dispersion for RNA-seq data with DESeq2. Genome Biol. 2014;15:550.

**SUPPLEMENTARY TABLES**

**Supplementary Table S1. Monoclonal antibodies (mAbs).**

| **Human Antibody** | **Clone** | **Company** |
| --- | --- | --- |
| CD3-APC-A750 | UCHT1 | Beckman Coulter |
| CD4-PC5.5 | 13B8.2 | Beckman Coulter |
| CD4-Krome Orange | 13B8.2 | Beckman Coulter |
| CD5-PC7 | BL1a | Beckman Coulter |
| CD8-Pacific Blue | B9.11 | Beckman Coulter |
| CD14-FITC | RMO52 | Beckman Coulter |
| CD19-APC-A750 | J3-119 | Beckman Coulter |
| CD45-Krome Orange | J33 | Beckman Coulter |
| CD45RA-Alexa Fluor 700 | 2H4LDH11LDB9 | Beckman Coulter |
| CD197(CCR7)-PE | G043H7 | Beckman Coulter |
| CD279(PD-1)-PC5.5 | PD1.3 | Beckman Coulter |
| HLA-DR-PC5.5 | Immu357 | Beckman Coulter |
| CD5-APC | L17F12 | BD Biosciences |
| CD160-Alexa Fluor 488 | BY55 | eBioscience |
| CD160-PE | BY55 | eBioscience |
| CD244-FITC | eBioDM244 | eBioscience |
|  |  |  |
| IL-10-PE | JES3-9D7 | eBioscience |
| T-bet-PE | eBio4B10 | eBioscience |
| Eomes-eFluor 660 | WD1928 | eBioscience |
| rat IgG1 κ isotype control-PE | eBRG1 | eBioscience |
| mouse IgG1 κ isotype control-eFluor 660 | P3.6.2.8.1 | eBioscience |
| CD279(PD-1)-Alexa Fluor 700 | EH12.2H7 | Biolegend |

**Supplementary Table S7.** **Highlighted dysregulated genes in T-CLL cells at progression.**

| **Gene^1^** | **Function** |
| --- | --- |
| *UBXN11* | Actine, microtubule and Rho-GTPase binding proteins |
| *CDC14A* |  |
| *HOOK2* |  |
| *NOA1* | Synthesis of nitric oxide |
| *ADAC8* | Fatty acids and amino acids catabolism |
| *NAPSA* |  |
| *FUT8* |  |
| *PRSS12* |  |
| *SLC2A3/GLUT3* | Glucose transporters |
| *SLC35A3* |  |
| *PTCD1* | RNA processing mechanisms |
| *NSUN4* |  |
| *FOSB* | Immune response and exhaustion |
| *JUN* |  |
| *PRSS12* |  |
| *FAM46C* |  |
| *NAPSA* |  |
| *TNS2* | Adhesion molecules |
| *FARP2* |  |
| *SPG7* | Maintenance of OXPHOS |
| *C8orf41* |  |
| *DDX23* |  |
| *ERMP1* |  |
| *SGPP2* | Synthesis of cellular components |
| *MGAT4B* |  |
| *DDX23* | RNA processing mechanisms |
| (1) Red: up-regulated genes; green: down-regulated genes |  |

**SUPPLEMENTARY FIGURES AND LEGENDS**

**
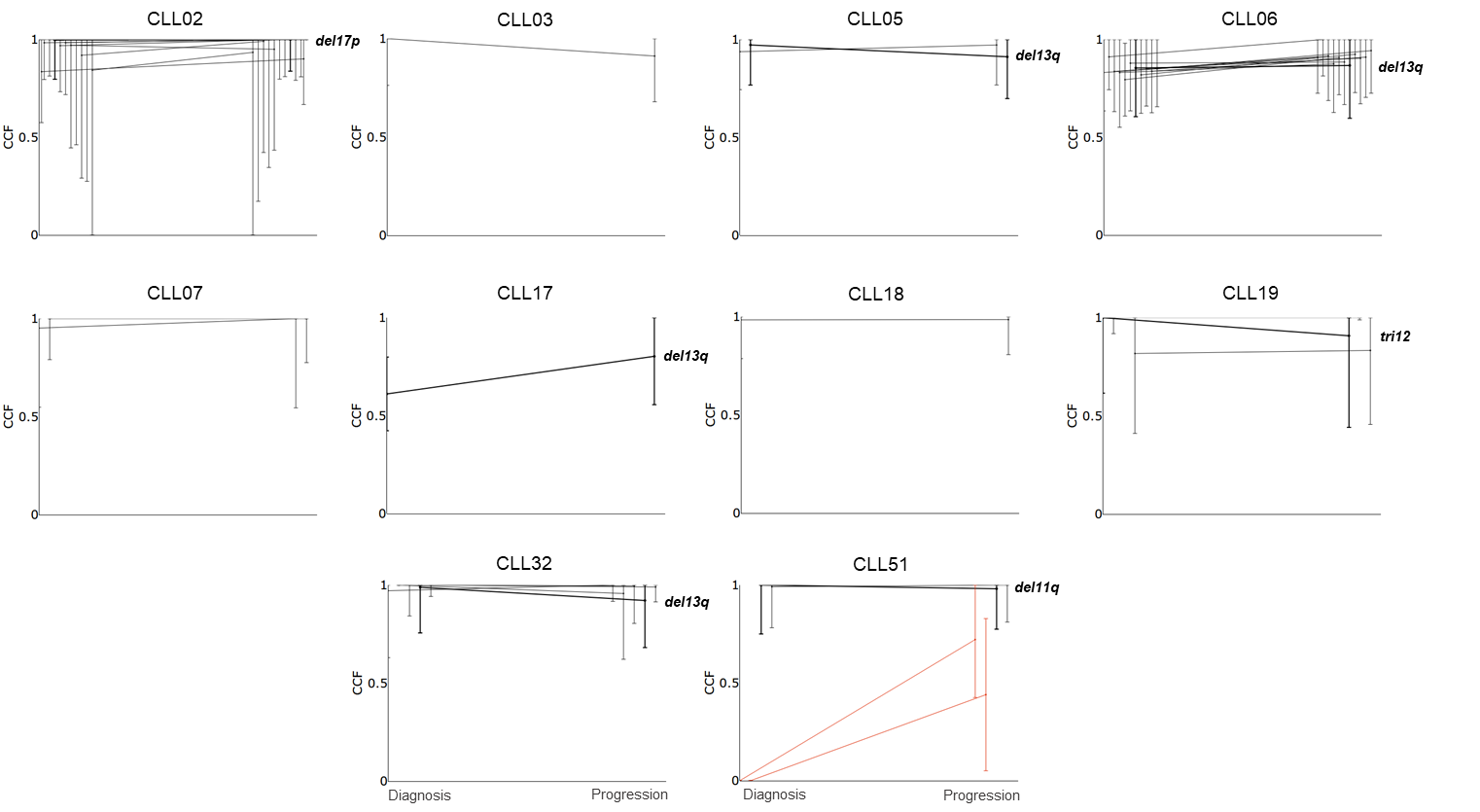
**

**Supplementary Figure S1. Longitudinal analysis of the CCF of CNVs from paired B-CLL cells at diagnosis and progression before treatment.** Comparison of the CCF with 95% CI for each CNV detected per patient (n=10) between diagnosis and progression. Significantly increased (red lines) and stable CCF (grey lines) are shown. Recurrent CNVs in CLL (del(13q), del(11q), del(17p) and tri(12)) are plotted with bold lines and labeled with CNV name: stable CCF (bold black) is shown.

**Supplementary Figure S2. CD8^+^ T-cell differentiation subsets and PD1 expression in CD8^+^ T cells from progressing and non-progressing CLL patients. a** CD4/CD8 ratio in progressing (n=19) and non-progressing patients (n=10) at diagnosis and progression or non-progression. **b** Absolute numbers of CD8^+^ T-cell differentiation subsets (naïve: CCR7^+^CD45RA^+^; central memory, CM: CCR7^+^CD45RA^-^; effector memory, EM: CCR7^-^CD45RA^-^ and EM CD45RA^+^, EMRA: CCR7^-^CD45RA^+^) in progressing (n=19) and non-progressing patients (n=10) at diagnosis and progression or non-progression. **c** Absolute numbers of PD1^+^CD8^+^ T cells in progressing (left, n=19) and non-progressing patients (middle, n=10) at diagnosis and progression or non-progression. Fold change of PD1^+^CD8^+^ T cells between time points comparing progressing and non-progressing patients (right). **d** Percentage of PD1^+^CD244^+^ CD8^+^ T cells in progressing (left, n=12) and non-progressing patients (middle, n=9) at diagnosis and progression or non-progression. Fold change of PD1^+^CD244^+^CD8^+^ T cells between time points comparing progressing and non-progressing patients (right). **e** Percentage of PD1^+^160^+^ CD8^+^ T cells in progressing (left, n=12) and non-progressing patients (middle, n=9) at diagnosis and progression or non-progression. Fold change of PD1^+^CD160^+^CD8^+^ T cells between time points comparing progressing and non-progressing patients (right). **f** Density plots of PD1, CD160 and CD244 coexpression in CD8^+^ T cells in representative patients at diagnosis and progression and at diagnosis and non-progression. Graphs show mean ± SEM or paired values (*P<0.05; **P<0.01; ***P<0.001; Wilcoxon matched paired test or Mann-Whitney test).

**
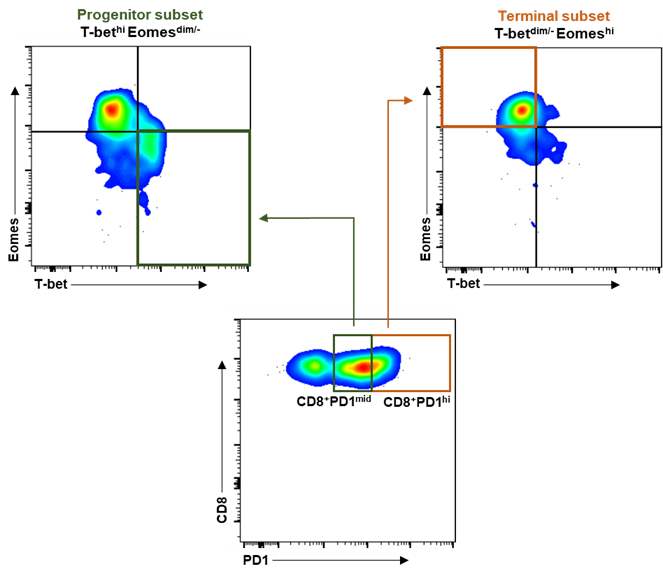
**

**Supplementary Figure S3.** **Flow cytometric analysis of progenitor and terminal CD8^+^ subsets.** Gating strategy followed for the identification of T-bet^hi^Eomes^dim/-^PD1^mid^ and T-bet^dim/-^Eomes^hi^PD1^hi^ CD8^+^ populations.

**Supplementary Figure S4.** **Co-expression of PD1 and CD244 in CD8^+^ T cells after co-culture with B-CLL cells. MDSCs in progressing and non-progressing CLL patients. a** Percentages of PD1^+^CD244^+^ cells out of CD8^+^ T cells from progressing (left) and non-progressing (right) CLL patients after stimulation with anti-CD3 and anti-CD28 for 7 days (grey dots) and in presence of B-HD cells (yellow dots) or B-CLL cells at the time of progression (red dots, n=10) or asymptomatic follow-up (blue dots, n=7) at the indicated T:B ratios. **b** Percentages of PD1^+^CD244^+^ cells out of CD8^+^ T cells from healthy age-matched donors (T-HD) after stimulation with anti-CD3 and anti-CD28 for 7 days (grey dots) and in presence of B-CLL cells at progression (bold red dots) or B-CLL cells at asymptomatic follow-up (bold blue dots) at the indicated T:B ratios. **c** Percentages of CD8^+^ T cells from CLL patients co-expressing PD1 and CD244 after stimulation with anti-CD3 and anti-CD28 for 7 days and in contact with B-CLL cells or separated by transwell inserts at 1:10 T:B ratio for 7 days (n=14).**d** Dot plots of IL-10^+^ B cells gated on CD19^+^CD5^+^ cells after 5 hours of leukocyte stimulation (PIB), or brefeldin A (BFA) as control, from one representative progressed and non-progressed patient. **e** Percentage of MDSCs (CD14^+^HLA-DR^low/-^) out of CD14^+^ cells in progressing (left, n=17) and non-progressing patients (middle, n=10) at diagnosis and progression or non-progression. Increment of MDSCs between time points comparing progressing and non-progressing patients (right). Graphs show mean ± SEM or paired values (*P<0.05; **P<0.01; ***P<0.001; ****P<0.0001; Wilcoxon matched paired test or Mann-Whitney test).
